# Supplementary material for: Quantifying cerebral contributions to pain beyond nociception
Source: Nat Commun. 2017 Feb 14;8:14211. doi: 10.1038/ncomms14211 (PMC5316889; doi:10.1038/ncomms14211)
Supplement: Supplementary Information — Supplementary Figures, Supplementary Tables and Supplementary References [file ncomms14211-s1.pdf]

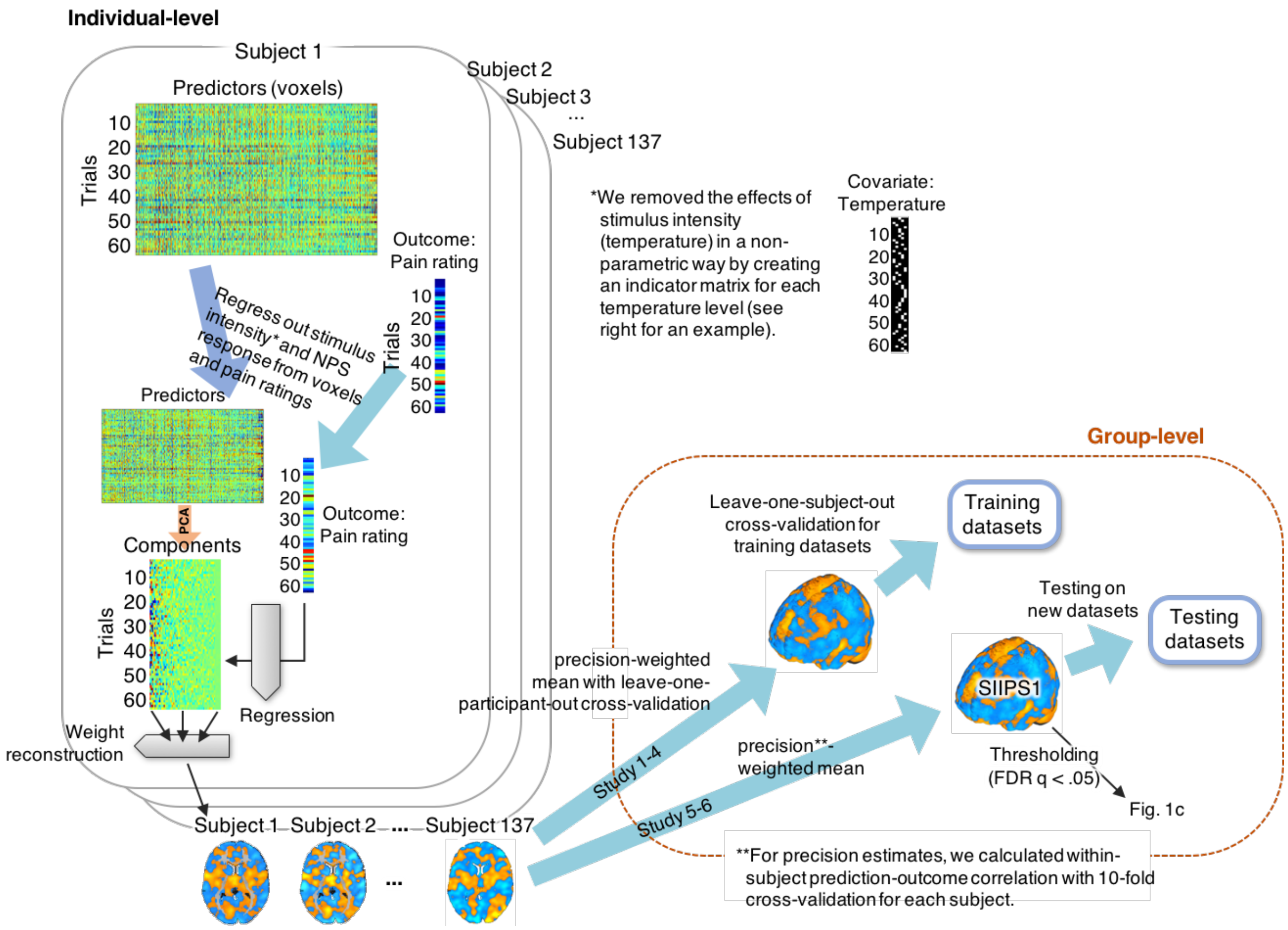

Supplementary Figure 1. Overview of the SIIPS1 development. The development of the SIIPS1 consisted of individual- and group-level analysis steps. **1) Individual-person level:** For each individual in the training datasets (Studies 1-4), we first regressed out stimulus intensity and the Neurologic Pain Signature (NPS)[1] response from single-trial estimates of brain activity and pain ratings. We removed the effects of stimulus intensity (temperature) in a nonparametric way by creating an indicator matrix for each temperature level. Then, we used principal component regression (PCR)[2] to predict residualized pain rating from residualized single-trial whole brain activity to obtain stable predictive models with high-dimensional, collinear predictors. **2) Group-level:** After we obtained predictive maps for all individuals, we constructed a group map using precision-weighted average. For precision estimates, we calculated prediction-outcome correlation with 10-fold cross-validation for each subject. For further analyses on the training datasets (Studies 1-4), we used leave-one-subject-out precision-weighted average maps to obtain unbiased results. For testing datasets (Studies 5-6), we used a precision-weighted average map based on all the data in the training datasets. For display (as presented in **Fig. 1c**), we conducted weighted *t*-test and thresholded the map with the false-discovery rate (FDR) correction at $q < .05$.

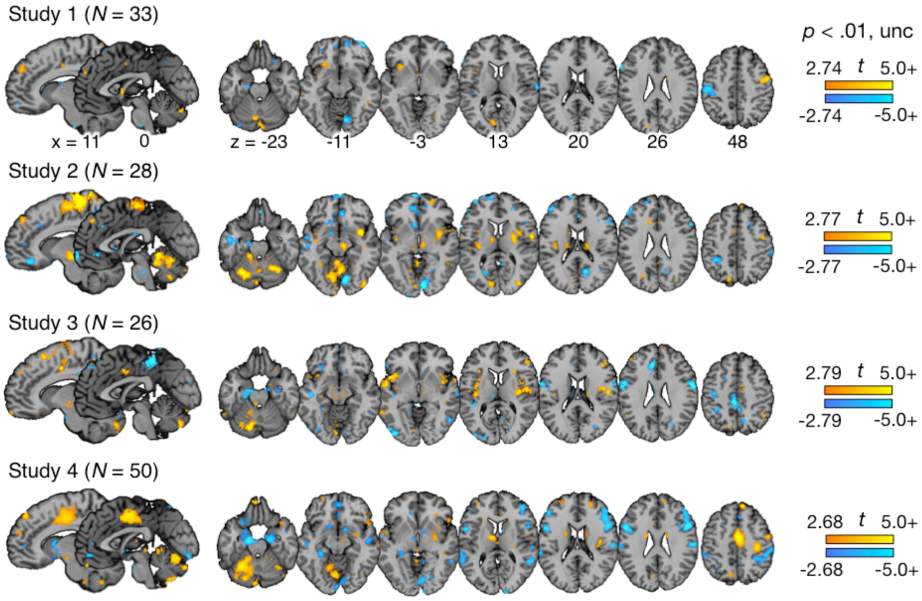

Supplementary Figure 2. Principal component regression (PCR) weight maps for each separate study in the training study set. Using individuals' PCR maps (see **Supplementary Figure 1**), we obtained precision-weighted average for each study. We used a weighted *t*-test and thresholded the maps for display at voxel-wise uncorrected $p < .01$.

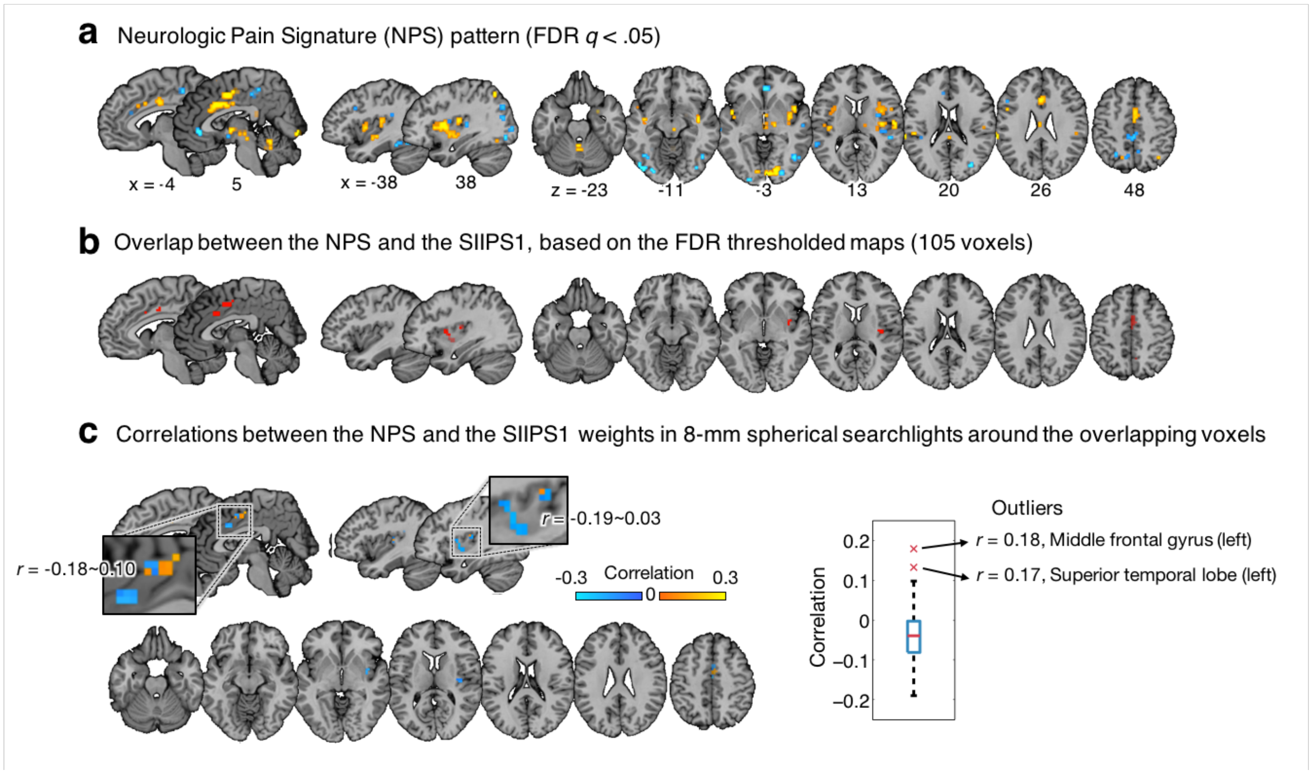

Supplementary Figure 3. Spatial pattern similarity between the SIIPS1 and the NPS[1]. (a) The NPS pattern, an *a priori* distributed pattern of fMRI activity that is sensitive and specific to physical pain, thresholded at $q < .05$, FDR corrected. **(b)** These 105 voxels had significant predictive weights for both the NPS and the SIIPS1 thresholded at $q < .05$, FDR corrected. **(c)** Correlation of spatial weights within 8-mm spherical regions around the overlapping voxels. For this analysis, we used unthresholded maps to include all the voxel weights within spherical regions. The spatial correlations between the NPS and SIIPS1 were low (mean $r = -0.037$, maximum correlation $r = 0.18$ in middle frontal gyrus).

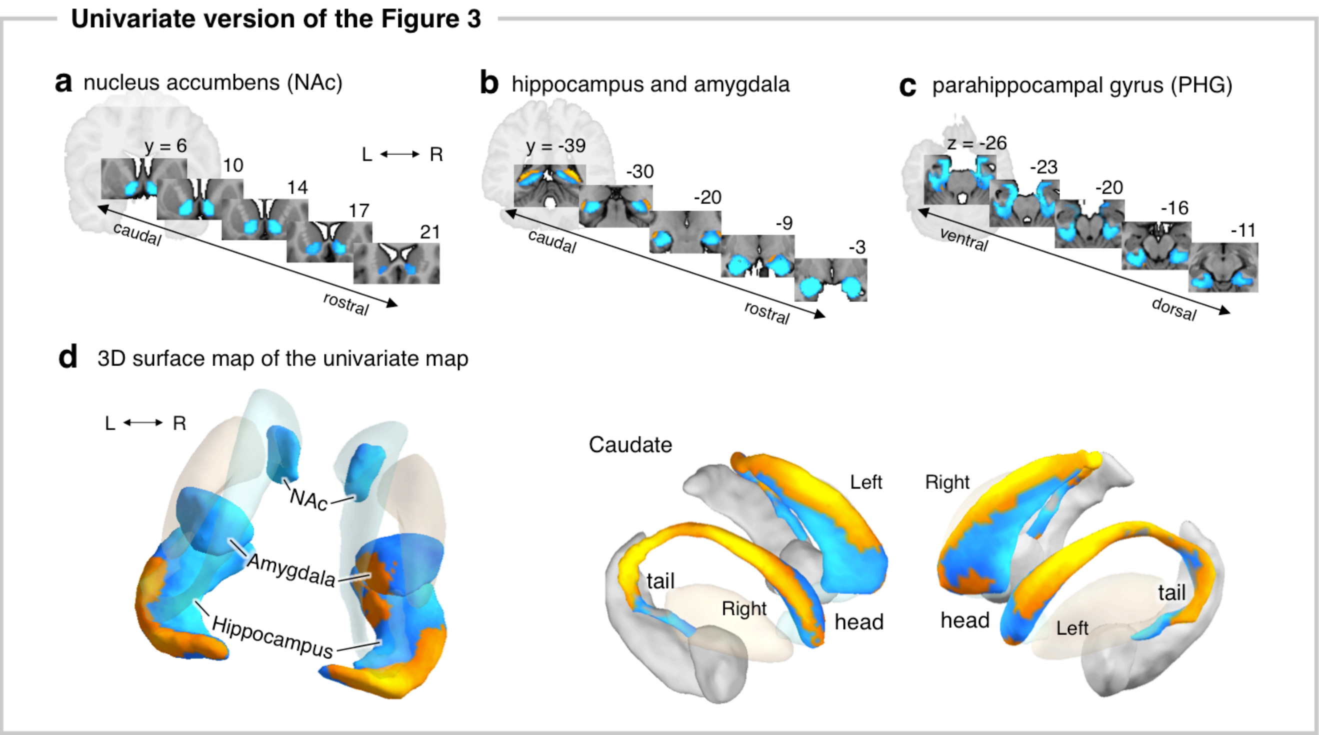

Supplementary Figure 4. The univariate version of the Figure 3. The univariate analysis did not show the fine-grained patterns of sub-regions' differential contributions that the multivariate analysis found, suggesting that the multivariate approach provides finer-grained and more sensible patterns about pro- and anti-pain sub-regions than the univariate approach.

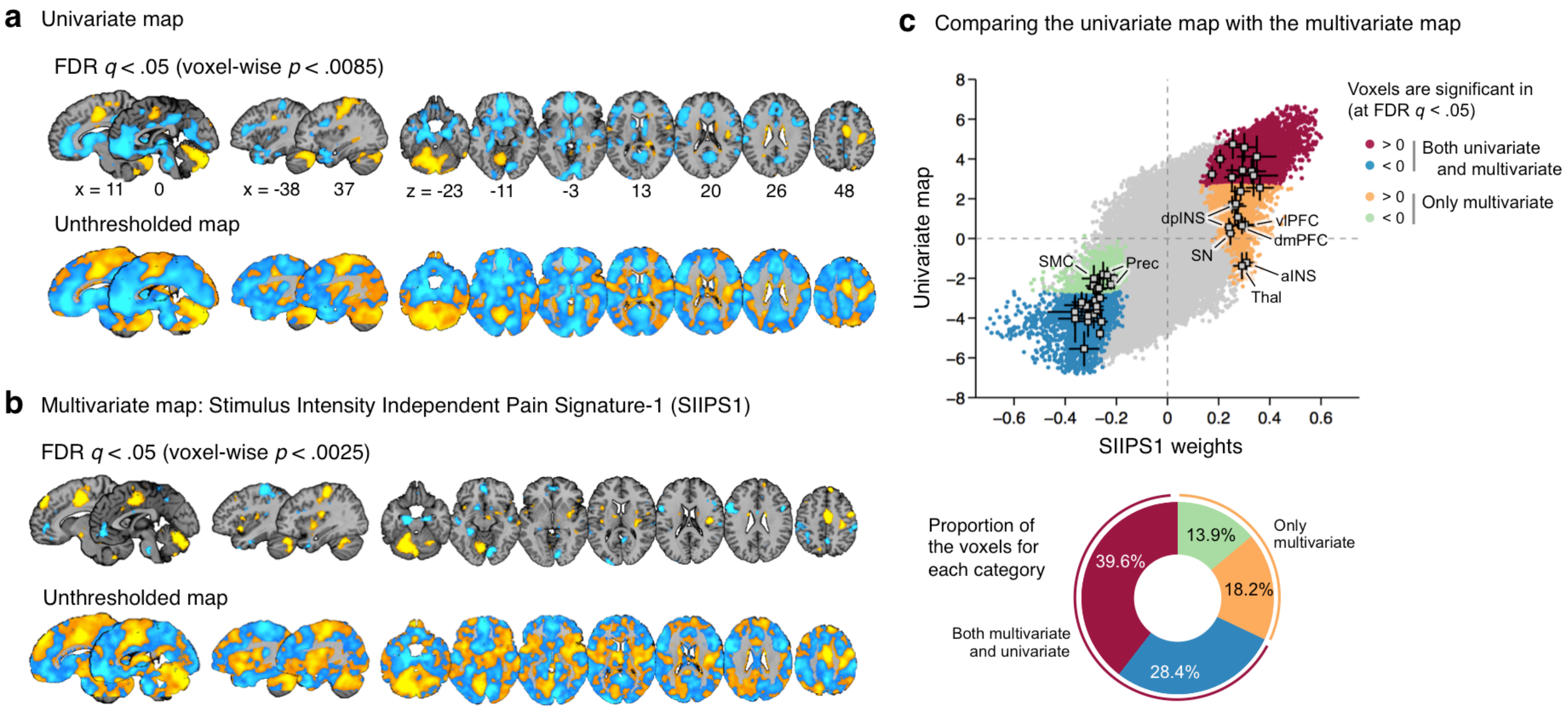

Supplementary Figure 5. Comparisons between the univariate vs. multivariate maps. (a) Univariate map. In individual-level analyses, we regressed pain ratings (x) on trial-by-trial fMRI activity (y) in each voxel, after regressing out the stimulus intensity (using multiple covariates to span the space of all possible intensity effects) and the NPS response. This yielded 137 maps of pain correlates (parameter effect maps). We performed one sample *t*-tests on these maps at the group level, treating participant as a random effect, and thresholded the map with False Discovery Rate (FDR) $q < .05$ correction for multiple comparisons (equivalent to voxel-wise $p < .0085$) for display purpose only. **(b)** The multivariate map: Thresholded and unthresholded SIIPS1. **(c)** A scatter plot of unthresholded voxel weights for the SIIPS1 versus the univariate map. Colored dots represent voxels that are significant in the multivariate map, and different colors indicate whether the voxel is significant in both multivariate and univariate maps or only in the multivariate map. The squares show the joint weights for regions significant in the SIIPS1 with ±1 SD error bars. The pie chart shows the proportion of the voxels that are significant in both multivariate and univariate maps or only in the multivariate map. INS, insular cortex; PFC, prefrontal cortex; Prec, precuneus; SMC, sensory motor cortex; SN, substantia nigra; Thal, thalamus; a, anterior; d, dorsal; l, lateral; m, medial; p, posterior; v, ventral. **Interpretation:** Most voxels show a high degree of agreement between the univariate and multivariate results,

indicating that they contribute positively or negatively to pain prediction whether tested independently (univariate) or jointly (multivariate; i.e., controlling for other regions). Some regions are associated with pain more strongly in the univariate than multivariate maps, suggesting that their association with pain is indirect and better explained by other brain regions[3]. This might point to a need to interpret univariate findings with caution. Some regions only appear in the multivariate map, suggesting that controlling for other regions reduces noise or unmasks significant relationships with pain[3].

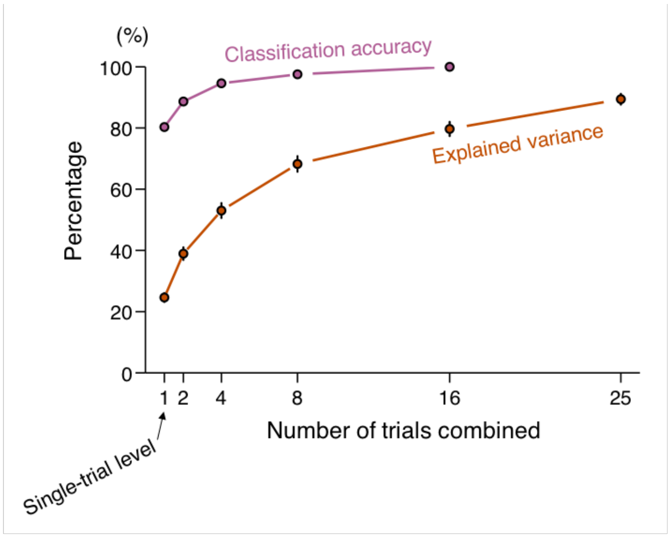

Supplementary Figure 6. Explained variance and classification accuracy as a function of the number of trials combined (Study 1-2, *N* = 61). Two fMRI signatures (the SIIPS1 and the NPS) together explained 25% variance in single trial-level pain ratings, which corresponded to 80.3% accuracy in the classification of high vs. low pain trials (top 30% vs. bottom 30% pain ratings). As more trials were combined (according to their pain rating bins), two fMRI signatures explained larger variance. Error bars represent standard errors of the mean (SEMs). We conducted this analysis with data from Studies 1 and 2 because these two studies had a large enough number of trials for the analysis. We note that the prediction of pain intensity and classification of which groups are more painful than others is unbiased, because the model has no prior information about which groups of trials are more painful than others.

Supplementary Table 1. Study demographics and prior publications

Study number	*N*	Sex	Ages Mean (SD)	Prior publications	Training/ Testing dataset
Study 1	33	22 F	27.9 (9.0)	Wager et al. 2013[1]; Woo et al., 2015[4]	Training
Study 2	28	10 F	25.2 (7.4)	Chang et al., 2015[5]; Krishnan et al., 2016[6]	Training (somatic pain); testing (vicarious pain)
Study 3	26	9 F	27.8 (7.5)	Wager et al., 2013[1]; Atlas et al., 2014[7]	Training
Study 4	50	27 F	25.1 (6.9)	Roy et al., 2014[8]	Training
Study 5	17	9 F	25.5	Atlas et al., 2010[9]	Testing
Study 6	29	16 F[a]	20.4 (3.3)[b]	New data	Testing
Total	183	93 F			Training n = 137<br>Testing n = 46

Note. [a] Sex of one participant is unknown; [b] Age of one participant is unknown. Training data are used in cross-validated training and testing analyses. Testing data were used to evaluate predictive accuracy and the mediation of psychological interventions only once the final signature was developed.

Supplementary Table 2. Task characteristics for studies

Study number	Stimulated locations	Number of intensity levels	Fixed vs. calibrated	Duration (seconds)	Rating Scale	Number of trials per subjects	Mean number of excluded trials (high VIFs)	Other experimental manipulations
Study 1	Arm	6 (44.3-49.3°C)	Fix	12.5	0-200 VAS[a]	97	6.8	Cognitive self-regulation to increase or decrease pain
Study 2	Arm, Foot	3 (46-48°C)	Fix	11	0-100 LMS[b]	81	6.1	Heat-predictive visual cues for low, medium, and high pain
Study 3	Arm	4 (PT/L/M/H)	Cal	10	0-10 VAS[c]	48	3.8	Masked emotional faces evenly crossed with temperature
Study 4	Arm	3 (46-48°C)	Fix	11	0-100 VAS[d]	48	5.7	Heat-predictive visual cues with a placebo manipulation
Study 5	Arm	4 (L/M/H)	Cal	10	0-10 VAS[c]	64	2.1	Heat-predictive auditory cues
Study 6	Arm	2 (L/H)	Cal	10	0-100 VAS[d]	64	1	Perceived control (making vs. observing cue choice), Expectancy (80% vs. 50% probabilities of low pain)

Note. Stimulus intensity levels: N, Non-painful warmth; PT, pain threshold; L, Low painful heat; M, Medium painful heat; H, High painful heat; Fix, Fixed; Cal, Calibrated; VAS, visual analog scale. LMS, labeled magnitude scale. VIF, variance inflation factor. [a] Pain vs. no-pain decision followed by 0-100 VAS for either warmth or pain rating. [b] 0, no sensation; 1.4, barely detectable; 6.1, weak; 17.2, moderate; 35.4, strong; 53.3, very strong; 100, strongest imaginable sensation. [c] 0, no sensation; 1, non-painful warmth; 2, low pain; 5, moderate pain; 8, maximum tolerable pain. [d] 0, no pain; 100, worst imaginable pain.

Supplementary Table 3. Acquisition parameters

Study number	Study location	Scanner details	EPI parameters	Voxel size (mm^3)	Acquisition parameters	Discarded volumes	Stimulus software	Analysis software
Study 1	Columbia	3T Phillips Achieva TX	TR = 2000 ms<br>TE = 20 ms<br>FOV = 224 mm<br>Matrix = 64 × 64<br>Flip angle = 72°	3.0 × 3.0 × 3.0	42 Slices<br>Interleaved<br>SENSE = 1.5	4	E-prime	SPM8
Study 2	CU Boulder	3T Siemens Tim Trio	TR = 1300 ms<br>TE = 25 ms<br>FOV = 220 mm<br>Matrix = 64 × 64<br>Flip angle = 50°	3.4 × 3.4 × 3.4	26 Slices<br>Interleaved<br>iPAT = 2	6	Matlab	SPM8
Study 3	Columbia	1.5T GE Signa TwinSpeed Excite HD	TR = 2000 ms<br>TE = 34 ms<br>FOV = 224 mm<br>Matrix = 64 × 64	3.5 × 3.5 × 4.0	29 Slices	5	E-prime	SPM5, 8
Study 4	CU Boulder	3T Siemens Tim Trio	TR = 1300 ms<br>TE = 25 ms<br>FOV = 220 mm<br>Matrix = 64 × 64<br>Flip angle = 75°	3.4 × 3.4 × 3.0	26 Slices<br>Interleaved<br>iPAT = 2	6	E-prime	SPM8
Study 5	Columbia	1.5T GE Signa TwinSpeed Excite HD	TR = 2000 ms<br>TE = 40 ms<br>FOV = 224 mm<br>Matrix = 64 × 64<br>Flip angle = 84°	3.5 × 3.5 × 4.5	24 Slices<br>T2*-weighted spiral in/out pulse	5	E-prime	SPM5
Study 6	CU Boulder	3T Siemens Tim Trio	TR = 1980 ms<br>TE = 25 ms<br>FOV = 220 mm<br>Matrix = 64 × 64<br>Flip angle = 75°	3.4 × 3.4 × 3.0	35 Slices<br>Interleaved<br>iPAT = 2	5	E-prime	SPM8

Note. TR, Time to repeat; TE, Time to echo; FOV, Field of view.

Supplementary Table 4. Regions that reliably contribute to the prediction of the SIIPS1 and mean correlations with noxious stimulus intensity

Regions	x	y	z	Number of voxels	Correlations with noxious stimulus intensity					
Regions with **positive** weights					Rank	*r*	*Fisher's z*	SE	*t*	*p*
Cerebellum, Left	-18	-66	-24	2546	5	0.211	0.223	0.015	14.58	0.0000
Middle cingulate cortex/Supplementary motor area	6	-6	48	1015	3	0.244	0.260	0.016	16.41	0.0000
Precentral gyrus, Right	36	-20	54	752	12	0.134	0.143	0.018	7.92	0.0000
Middle insula/Dorsal posterior insula/Putamen, Right	34	-10	8	440	4	0.224	0.236	0.014	17.26	0.0000
Cerebellum, Right	34	-56	-30	335	10	0.167	0.176	0.015	11.53	0.0000
Dorsal medial prefrontal, Right	12	50	40	218	28	0.003	0.004	0.012	0.28	0.7774
Superior parietal lobe, Right	26	-42	66	123	7	0.177	0.183	0.013	14.27	0.0000
Cerebellum (Vermis)	4	-64	-46	98	16	0.103	0.107	0.014	7.63	0.0000
Central opercular cortex, Left	-56	0	4	96	2	0.260	0.275	0.014	19.73	0.0000
Central opercular cortex, Right	58	2	6	51	1	0.270	0.286	0.014	20.15	0.0000
Superior parietal lobe, Left	-22	-42	64	46	17	0.093	0.096	0.013	7.33	0.0000
Anterior insula, Left	-38	14	-8	43	11	0.153	0.160	0.013	12.15	0.0000
Caudate, Right	18	-2	24	30	18	0.082	0.086	0.014	6.05	0.0000
Middle insula, Left	-36	8	10	28	6	0.201	0.211	0.014	15.25	0.0000
Middle temporal gyrus, Right	70	-20	-14	24	32	-0.017	-0.017	0.013	-1.34	0.1815
Thalamus, Left	-10	-6	10	22	14	0.122	0.127	0.014	9.22	0.0000
Inferior frontal gyrus, Right	54	24	-6	20	13	0.133	0.138	0.013	10.40	0.0000
Dorsal posterior insula, Left	-38	-16	12	20	8	0.171	0.177	0.013	13.81	0.0000
Substantia nigra, Right	10	-8	-12	19	15	0.118	0.123	0.014	8.93	0.0000
Caudate, Left	-18	-2	24	19	19	0.078	0.081	0.014	5.77	0.0000
Precuneus, Left	-12	-70	46	18	22	0.054	0.056	0.013	4.28	0.0000
Regions with **negative** weights										
Sensorymotor cortex, Left	-40	-28	58	888	41	-0.047	-0.048	0.015	-3.18	0.0017
Hippocampal/Parahippocampal regions, Left	-16	-20	-24	726	42	-0.055	-0.056	0.014	-4.01	0.0001
Sensorymotor cortex, Right	56	-26	42	369	20	0.060	0.063	0.014	4.49	0.0000
Dorsal lateral prefrontal, Left	-52	10	26	356	25	0.031	0.032	0.013	2.44	0.0155
Lingual gyrus, Right	12	-78	-8	337	26	0.019	0.020	0.014	1.43	0.1540
Ventromedial prefrontal	2	48	-6	316	43	-0.057	-0.058	0.014	-4.24	0.0000
Hippocampus, Right	24	-8	-28	246	40	-0.046	-0.047	0.014	-3.31	0.0011
Temporal pole, Right	32	8	-40	118	30	-0.008	-0.008	0.014	-0.61	0.5406
Cuneus, Right	10	-52	8	111	38	-0.037	-0.037	0.015	-2.48	0.0142
Dorsal lateral prefrontal, Right	52	12	26	105	21	0.055	0.056	0.013	4.23	0.0000
Middle occipital gyrus, Left	-22	-98	12	101	36	-0.035	-0.035	0.013	-2.64	0.0091
Superior temporal gyrus, Left	-60	-30	6	98	27	0.013	0.015	0.015	1.03	0.3051
Ventral striatum, Left	-14	14	-14	92	29	-0.004	-0.004	0.013	-0.31	0.7606
Precuneus, Left	-6	-46	64	90	23	0.038	0.040	0.016	2.48	0.0142
Precuneus, Right	4	-54	48	83	35	-0.030	-0.030	0.015	-2.08	0.0393
Temporal pole, Left	-32	14	-44	62	34	-0.027	-0.027	0.013	-2.03	0.0437
Superior temporal gyrus, Right	54	-12	-10	58	39	-0.043	-0.043	0.014	-3.15	0.0019
Middle temporal gyrus, Left	-52	-16	-10	45	44	-0.061	-0.062	0.013	-4.68	0.0000
Superior parietal lobe Left	-34	-56	52	39	37	-0.035	0.035	0.012	-2.84	0.0051
Inferior temporal gyrus, Left	-38	-4	-32	27	31	-0.011	-0.011	0.014	-0.78	0.4363
Secondary somatosensory (SII), Right	62	-22	18	21	9	0.171	0.178	0.014	13.16	0.0000
Precentral gyrus, Middle	-6	-24	52	20	33	-0.023	-0.023	0.017	-1.38	0.1705
Ventral striatum, Right	16	10	-12	7	24	0.034	0.035	0.013	2.62	0.0095

Note. The reported x, y, z coordinates are in MNI space. Rank shows the rank numbers from the region with the highest correlation value. *r* is the mean correlation between the local pattern expression using absolute pattern weights and noxious input intensity across 183 participants from 6 independent studies. We used absolute pattern weights in this analysis to make correlation values easy to interpret; positive correlations mean positive relationships between the region activity and stimulus intensity, and negative correlations indicate negative relationships between the region activity and stimulus intensity. *z* represents Fisher's *z*-transformation of *r*, and SE is standard error of the *z* values. *t*- and *P*-values are calculated

from t-tests on the z values across participants. Red indicates regions that are significantly correlated with noxious input intensity (Family-wise error rate $p < .05$ using Bonferroni correction), and gray indicates regions independent of noxious input intensity.

Supplementary Table 5. Path coefficients for mediation analyses of psychological modulation effects

Psychological manipulations	Mediators	Path *a* (X→M)			Path *b* (M→Y)			Path *a*×*b* (X→M→Y)			Correlation(*a.b*)[d]		
		β_1	SE	P	β_2	SE	P	$\beta_1\beta_2$	SE	P	*R*		
Two-path mediation													
Expectancy (Study 5)[a]	SIIPS1	-0.162	0.149	0.209	0.102	0.076	0.209	-0.044	0.025	**0.073**	-0.133		
	NPS	-0.273	0.101	**0.005**	0.087	0.045	**0.050**	-0.004	0.012	0.682	-0.263		
Expectancy (Study 6)[b]	SIIPS1	-0.035	0.023	0.121	2.117	0.355	**0.001**	-0.063	0.030	**0.035**	-0.349		
	NPS	-0.062	0.022	**0.004**	1.499	0.384	**0.000**	-0.060	0.028	**0.031**	0.036		
		Path b_1 (X→M1)			Path b_2 (M1→M2)			Path b_3 (M2→M3)			Path $b_1b_2b_3$ (X→M1→M2→Y)		
Three-path mediation		β_1	SE	P	β_2	SE	P	β_3	SE	P	$\beta_1\beta_2\beta_3$	SE	P
Perceived control (Study 6)[c]	SIIPS1	4.101	1.829	**0.008**	-0.002	0.001	**0.041**	2.354	0.366	**0.001**	-0.007	0.003	**0.037**
	NPS	4.308	1.830	**0.003**	-0.001	0.001	0.581	1.584	0.380	**0.000**	0.000	0.006	0.965

Note. [a] For X, low vs. high pain cues coupled with medium heat pain (i.e., LM vs. HM) were coded as 1 and -1, respectively. [b] For X, high vs. low expectation about receiving low pain (i.e., HE vs. LE) were coded as 1 and -1. [c] For X, high vs. low perceived control (i.e., HC vs. LC) were coded as 1 and -1, and self-reported perceived control was entered as M1 (first mediator). [d] Empirical Bayes weighted correlation between Path *a* and *b*. Note that Paths *a* and *b* did not always show significant effects even when the mediation effects (Path $a \times b$) were significant across different studies. This is a common phenomenon in multilevel mediation analyses when Paths *a* and *b* covary [9,10]. The significant mediations driven by the covariation of Paths *a* and *b* indicate that even though the Path *a* and *b* effects are heterogeneous across people (in other words, Paths *a* and *b* could be negative for some people, and positive for other people), Path $a \times b$ (the mediation effects) is consistent across people. Therefore, how the SIIPS1 works for each person could differ, even though the SIIPS1 is a significant mediator across people and studies.

Reference

1. Wager, T. D. *et al.* An fMRI-based neurologic signature of physical pain. *N. Engl. J. Med.* **368,** 1388–1397 (2013).
2. Hastie, T., Tibshiran, R. & Friedman, J. *The Elements of Statistical Learning: Data Mining, Inference, and Prediction*. (Springer, 2003).
3. Woo, C.-W. & Wager, T. D. in *Advances in Computational Psychophysiology* 18–21 (Science /AAAS, 2015).
4. Woo, C.-W., Roy, M., Buhle, J. T. & Wager, T. D. Distinct brain systems mediate the effects of nociceptive input and self-regulation on pain. *PLoS Biol.* **13,** e1002036 (2015).
5. Chang, L. J., Gianaros, P. J., Manuck, S. B., Krishnan, A. & Wager, T. D. A Sensitive and Specific Neural Signature for Picture-Induced Negative Affect. *PLOS Biol.* **13,** e1002180 (2015).
6. Krishnan, A. *et al.* Somatic and vicarious pain are represented by dissociable multivariate brain patterns. *eLife* **5,** e15166 (2016).
7. Atlas, L. Y., Lindquist, M. a, Bolger, N. & Wager, T. D. Brain mediators of the effects of noxious heat on pain. *Pain* **155,** 1632–48 (2014).
8. Roy, M. *et al.* Representation of aversive prediction errors in the human periaqueductal gray. *Nat. Neurosci.* **17,** 1607–1612 (2014).
9. Atlas, L. Y., Bolger, N., Lindquist, M. a & Wager, T. D. Brain mediators of predictive cue effects on perceived pain. *J. Neurosci. Off. J. Soc. Neurosci.* **30,** 12964–77 (2010).
10. Kenny, D. a., Korchmaros, J. D. & Bolger, N. Lower level mediation in multilevel models. *Psychol. Methods* **8,** 115–128 (2003).
